# Enhanced Flavonoid Accumulation Reduces Combined Salt and Heat Stress Through Regulation of Transcriptional and Hormonal Mechanisms

**DOI:** 10.3389/fpls.2021.796956

**Published:** 2021-12-21

**Authors:** Rahmatullah Jan, Nari Kim, Seo-Ho Lee, Muhammad Aaqil Khan, Sajjad Asaf, Lubna, Jae-Ryoung Park, Saleem Asif, In-Jung Lee, Kyung-Min Kim

**Affiliations:** ^1^Division of Plant Biosciences, School of Applied Biosciences, College of Agriculture and Life Science, Kyungpook National University, Daegu, South Korea; ^2^Coastal Agriculture Research Institute, Kyungpook National University, Daegu, South Korea; ^3^Natural and Medical Science Research Center, University of Nizwa, Nizwa, Oman; ^4^Department of Botany, Garden Campus, Abdul Wali Khan University, Mardan, Pakistan

**Keywords:** flavanol 3-hydroxylase, kaempferol, quercetin, oxidative stress, transgenic plants, physiological

## Abstract

Abiotic stresses, such as salt and heat stress, coexist in some regions of the world and can have a significant impact on agricultural plant biomass and production. Rice is a valuable crop that is susceptible to salt and high temperatures. Here, we studied the role of flavanol 3-hydroxylase in response to combined salt and heat stress with the aim of better understanding the defensive mechanism of rice. We found that, compared with wild-type plants, the growth and development of transgenic plants were improved due to higher biosynthesis of kaempferol and quercetin. Furthermore, we observed that oxidative stress was decreased in transgenic plants compared with that in wild-type plants due to the reactive oxygen species scavenging activity of kaempferol and quercetin as well as the modulation of glutathione peroxidase and lipid peroxidase activity. The expression of high-affinity potassium transporter (*HKT*) and salt overly sensitive (*SOS*) genes was significantly increased in transgenic plants compared with in control plants after 12 and 24 h, whereas sodium-hydrogen exchanger (*NHX*) gene expression was significantly reduced in transgenic plants compared with in control plants. The expression of heat stress transcription factors (HSFs) and heat shock proteins (HSPs) in the transgenic line increased significantly after 6 and 12 h, although our understanding of the mechanisms by which the *F3H* gene regulates *HKT*, *SOS*, *NHX*, *HSF*, and *HSP* genes is limited. In addition, transgenic plants showed higher levels of abscisic acid (ABA) and lower levels of salicylic acid (SA) than were found in control plants. However, antagonistic cross talk was identified between these hormones when the duration of stress increased; SA accumulation increased, whereas ABA levels decreased. Although transgenic lines showed significantly increased Na+ ion accumulation, K+ ion accumulation was similar in transgenic and control plants, suggesting that increased flavonoid accumulation is crucial for balancing Na+/K+ ions. Overall, this study suggests that flavonoid accumulation increases the tolerance of rice plants to combined salt and heat stress by regulating physiological, biochemical, and molecular mechanisms.

## Introduction

The increase in global warming is causing a worldwide increase in abiotic stress to plants, including an increase in its intensity and duration, which can have a devastating effect on the growth, yield, and quality of crops (Pandey et al., 2017). Salinity, drought, and high temperature are common abiotic stresses that cause many physiological and molecular changes in plants that can lead to yield losses (Ahuja et al., 2010). The responses of plants to such combined stresses involve complex mechanisms, including multimolecular signaling pathways that control further transcriptional and hormonal responses, as well as metabolic pathways that potentially interact (Atkinson et al., 2013). In recent decades, many researchers have studied the responses of plants to abiotic stresses, thereby increasing our knowledge on the subject. Several studies have focused on plant responses to a single stress (Hirayama and Shinozaki, 2010; Chew and Halliday, 2011). Most agricultural land is exposed to combined stresses; in particular, combined salt and high temperature stresses can simultaneously regulate the responses of plants to both stresses (Kuznetsov and Shevyakova, 1997). Researchers have suggested that combinations of abiotic stresses are unique stresses in themselves and should be studied independently of their components (Mittler and Blumwald, 2010). Salinity and high temperature are two of the main constraints for the growth of food resources; therefore, adaptation strategies are required for the development of agronomic traits that increase the stress tolerance of plants (Francini and Sebastiani, 2019). Development of transgenic plant is a powerful tool to address various agronomic traits including breeding increased tolerance against biotic stress and abiotic stress (Lavania et al., 2015).

Rice is considered a salt-sensitive plant; indeed, salinity affects almost all growth stages and the development of rice. Salt stress inhibits seed germination, seedling growth, root and shoot growth, root and shoot fresh and dry weight, tiller and spikelet number, and leaf size and productivity (Reddy et al., 2017). The response of rice toward salinity stress depends on the plant’s stage of development; young seedlings are most sensitive to salt stress. Roots and shoots are both sensitive to salt stress; however, Misra et al. (1996) showed that increasing NaCl concentration increased the damage to the roots of mung beans compared with the damage inflicted to the shoots, while also increasing the number of lateral roots and root thickness. Under saline conditions, plants are unable to take up sufficient water, and this leads to osmotic stress, which is similar to physiological drought; consequently, the plant loses turgidity due to low osmotic potential (Verslues et al., 2006). Because of osmotic stress and ion injury, plants increase their uptake of Na, Mg, and Ca, which can cause nutritional imbalance that leads to various types of toxicity (Hanumantharao et al., 2016). High concentrations of salt can increase the hypersensitive response and H_2_O_2_ generation in both the roots and shoots (Saha et al., 2010). Salt stress also increases chlorosis and necrosis disease while reducing chlorophyll and carotenoid content in plants (Gulati and Jaiwal, 1993; Wahid et al., 2004). The chlorophyll contents depends on plant physiological responses and their ability to stress tolerance (Siddiqui et al., 2015).

Among the various phytohormones, abscisic acid (ABA) and salicylic acid (SA) are considered to be the most important hormones associated with abiotic stress. The cross talk between ABA and SA has been the subject of various studies, but the relationship of these two hormones in response to combined salt and heat stress has yet to be elucidated. ABA and SA have an antagonistic relationship in response to certain biotic and abiotic stresses (Alazem et al., 2019); however, some integrative effects have been reported with changes to some specific responses. ABA plays a major role in mitigating stress effects; it is a first line of defense that is synthesized in the roots to mitigate the inhibitory effect of salinity (Han et al., 2015; Khan et al., 2021a). Likewise, SA responds to abiotic stress and can reduce the inhibitory effect of salt stress by regulating biological and biochemical processes (Ashraf et al., 2010; Khan et al., 2015). Although the exact cross-talk mechanism is not yet known, it has been shown that the exogenous application of ABA decreases SA levels, whereas some studies suggest that ABA inhibits the activity of SA-glucosyltransferase and can increase SA levels (Liu et al., 2006; Szalai et al., 2011). Increased levels of ABA during abiotic stress are involved in the generation of reactive oxygen species (ROS), which in turn induce the antioxidant defense system (Jiang and Zhang, 2002; Khan et al., 2021b). Besides salt stress, SA and ABA are also involved in high-temperature stress (Khan et al., 2020b). Accumulation of SA increases in *brassica juncea* seedlings in response to heat, whereas ABA levels increase with short-term heat treatment in *nicotiana tabacum* and *pisum sativum* plants (Larkindale and Huang, 2005; Li et al., 2015). In contrast, high temperature does not cause any change in ABA accumulation in *solanum lycopersicu*m plants (Bray, 1991). Heavy metal-treated rice plants show decreased biosynthesis of both ABA and SA, which suggests that low levels of ABA and SA improve the antioxidant capacity of plants, which in turn induces the accumulation of protective compounds that can enhance the tolerance of plants to abiotic stress (Khan et al., 2020a).

For normal physiological activity, plants need to maintain a high K+ and Na+ ratio in their cytosol. However, under saline conditions, K+ and Na+ alterations can cause an imbalance in this ratio due to the increased concentration of Na+ in soil, which leads to the passive transport of Na+ to the cytosol (Blumwald et al., 2000). A high concentration of Na+ decreases the accumulation of K+ and increases osmotic pressure, which leads to oxidative damage and inhibited productivity, growth, and other metabolic activities (James et al., 2011). Benlloch-González et al. (2017) reported that heat stress reduces K+ efflux in the roots of *olea europaea* trees. However, a recent report showed that an increase in K+ content in berries was correlated with increased temperature (Chérel and Gaillard, 2019). Na+ toxicity is an early mechanism of cell damage in salt-sensitive plants, whereas K+ is an important ion (Kader et al., 2006). To maintain the optimal ratio of Na+ and K+, plants have developed some essential mechanisms that include limiting the absorption of Na+, reducing transportation, storing Na+ in the leaf sheath, and cycling Na+ from shoots to roots (Tian et al., 2010). *HKT*, *NHX*, and *SOS* genes play an essential role in the transport of ions such as Na, K, and Ca (Shi et al., 2002; Garciadeblás et al., 2003; Rodríguez-Rosales et al., 2009). SOS transporter transports intracellular Na+ out of the cell or transports it into the root xylem *via* HKT, which reduces Na+ toxicity in the plant stem (Shi et al., 2002). In some cases, Na+ is sequestrated into the vacuole in the tonoplast *via* NHX (Apse et al., 1999). Besides regulating ion transporter genes, plants also respond to high temperature, salt, and drought stresses by regulating transcription factor networks such as heat stress transcription factors (HSFs). HSFs play an important part in the regulation of stress-responsive genes, such as *HSP* genes, in response to certain abiotic stresses including salt and heat stress (Guo et al., 2016). *HSP* is found in bacteria, yeast, and multicellular plants and localized to different compartments of cell, which play a decisive roles in cell adaptation to heat stress (Mishra and Grover, 2016). Previous reports show that HSFA1 transcription factor is upregulated in response to salt stress, osmotic pressure, and oxidative stress at the seedling stage (Ogawa et al., 2007; LIU et al., 2011). Over-expression of HSFA1 in *glycine max* has been shown to increase heat tolerance due to the induction of *HsP70* and *HsP22* genes (Zhu et al., 2006). In contrast, a HSFA2-knockout mutant line was completely unable to mitigate heat tolerance; however, this mechanism is not specific to HSFA2 because other HSFs can also exert positive feedback on other HSFs (Jacob et al., 2017). The expression levels of these genes depend on the pattern of applied stress. In general, the expression pattern of a gene differs when stresses are combined or when the component stresses are applied individually (Rizhsky et al., 2002; Mittler, 2006).

In response to long-term abiotic stress, plants have evolved to activate their antioxidant machinery, e.g., antioxidant enzymes (glutathione peroxidase and malondialdehyde) and flavonoids (kaempferol and quercetin), to mitigate the cytotoxic effects of generated ROS. It has been reported that flavonoid molecules enhance salt stress tolerance through the scavenging of free radicals, a process that reduces oxidative stress (Chandran et al., 2019). Previous studies have shown that flavonoid biosynthesis is induced by salt stress and enhances plant quality (Yan et al., 2014; Chen et al., 2019). Quercetin is an important flavonoid that has effective antioxidant properties; it plays a key role in mitigating oxidative stress as well as enhancing salinity and high-light tolerance in *Arabidopsis thaliana* and *Ligustrum vulgare* (Agati et al., 2011; Chan and Lam, 2014). It has also been reported that the accumulation of kaempferol and quercetin is enhanced during combined heat and salt stress; however, this regulation does not occur with salt stress alone (Martinez et al., 2016). Bian et al. (2020) found that the *HSFB2b* gene enhances flavonoid biosynthesis, which improves salt tolerance in *glycine max* plants. Similarly, we previously reported that flavanol 3-hydroxylase is a key regulator of flavonoid biosynthesis that participates in the responses to several stresses including UV stress and salinity (Liu et al., 2013). In *solanum lycopersicu*m, during combined heat and salt stress, the accumulation of flavonoids is enhanced due to the upregulation of the *F3H* gene; however, *F3H* was downregulated with salt stress alone in *solanum lycopersicu*m plants, whereas *F3H* transcript levels increased in *Reaumuria trigyna* in response to salt stress (Zhang et al., 2014; Martinez et al., 2016). Indeed, it was previously reported that about 60% of the total expressed transcripts cannot be predicted from a single stress, which highlights the importance of studying combined stresses during plant breeding and the engineering of tolerant plants grown in abiotic environments (Yamaguchi and Blumwald, 2005; Mittler, 2006). As F3H has been shown to be involved in various stress responses due to the regulation of flavonoid biosynthesis, we investigated the physiological responses and phenotypic variation of rice F3H transgenic plants in response to combined salt and heat stress. Overall, the aim of our study was to understand the response mechanisms of rice F3H transgenic and wild plants to combined salt and heat stress. Our study will help improve understanding of the role of F3H overexpression in the regulation of flavonoids, hormones, antioxidants, and Na+ and K+ uptake in plants subjected to salt and heat stress combined.

## Materials and Methods

### Plant Material, Growth Conditions, and Phenotypic Evaluation

In the current experiment, we used nagdong rice cultivar, we used F3H gene overexpressor line of nogdang cultivar (OxF3H; Jan et al., 2020) and nagdong wild-type plants. The seeds were pretreated with fungicides overnight and then washed three times with double-distilled water. The clean seeds were then soaked for 3 days in water at 32°C in the dark, and the water was changed every day following Jan et al. (2020). During the incubation period, the seeds sprouted and were transferred to autoclaved soil, after which they were kept in the dark again for 3 days. After successful growth, the seedlings were exposed to light and kept in a greenhouse for further experimentation.

### Experimental Design

We selected three groups of nagdong rice cultivar to evaluate the effect of combined salt and heat stress on rice plants. The control group, i.e., the wild-type control group (Wt-cont), was not exposed to stress. One treatment group, wild-type treated (Wt-t), was exposed to stress conditions. The other treatment group contained F3H overexpressor (OxF3H) plants was exposed to stress condition; thus, the group was named OxF3H-treated (OxF3H-t). We used only one transgenic line (as reported in our previous study) for comparison with the wild-type control and treated groups. The plants were grown for 1 month under normal conditions (temperature 28–30°C, light 16/8 h) in a greenhouse with optimized conditions. After 1 month, Wt-t and OxF3H-t plants were treated with 150-mM NaCl in growth chamber maintaining 40°C temperature, whereas Wt-cont plants were kept under normal conditions. Samples were collected for transcriptional, hormonal, and other physiological analyses; however, phenotypic data such as root/shoot length, leaf width, and leaf infected area were collected after 1 week of continuous exposure to salt and heat stress.

### RNA Isolation and qRT-PCR

Leaf samples were collected for total RNA extraction from the plants in all three groups in triplicate after 0, 1, 6, 12, and 24 h of stress exposure. RNA extraction, cDNA synthesis, and qRT-PCR were performed following the methods of Jan et al. (2020). The primer and accession number for each gene are listed in Table 1. We used RNeasy Plant Mini Kits (50) from Qiagen for total RNA extraction, and qPCRBIO and qPCRBIO SYBR Green Kits from PCR Biosystems for cDNA synthesis and qRT-PCR, respectively. Actin was used as a reporter gene. An Eco Real-Time (Illumina, Singapore) machine was used to complete qRT-PCRs using a 20-μL reaction volume (10 μl of SYBR green, 7 μl of ddH_2_O, 1 μl of template DNA, and 1 μl of primer).

**Table 1:** Gene name, primers, and accession number.

S. no.	Gene name	Primer sequence	Accession number
1	F3H	ATGGCGCCGGTGGCCACGAC (sense) GATGAATCCGCCCTTCTTGCCG (antisense)	MT980835.1
2	HKT	GATCCCGCAGATTCTAGCAG (sense) GGCAATTCGGATTTTCAGTG (antisense)	LC533374
3	HSF	TATGCCAAATGGTCAAGGTC (sense) CAGGCAAAGAGAATACACCC (antisense)	XM_015774935
4	HSP	GCGTCAAGAAGGAGGAGGT (sense) GTGCCACTTGTCGTTCTTGT (antisense)	XM_015775444
5	NHX	CGTGGTGATCTTGCTGATGA (sense) TCATCTTCCTCCATGGCTCTG (antisense)	KY752545
6	SOS	ATGGACAATCCCGAGGCGGA (sense) ACAGCCAGAAGAAGATCAGG (antisense)	KY752550
7	Actin	TCACCATCGGAGCAGAAAG (sense) AAAAGATGGCTGGAAGAGCA (antisense)	XM_015785964.2

### Quantification of Endogenous SA and ABA

We quantified both SA and ABA to determine the cross talk between the two in response to combined salt and heat stress. Leaves from the control, Wt-t, and OxF3H-t plants were collected after 0, 3, 6, 12, and 24 h, and the freeze-dried samples were ground in liquid nitrogen into a fine powder. Quantification and extraction of both SA and ABA were performed using previously published protocols (Jan et al., 2019). For SA analysis, 0.3 g sample of freeze-dried fine powder was sequentially extracted with 2 ml of 90 and 100% methanol (MeOH) and then centrifuged for 20 min at 1,000 rpm. The supernatant was then collected, and the MeOH of the supernatant was dried in a vacuum centrifuge. The dried pellets of the sample were resuspended in 3 ml of 5% trichloroacetic acid (TCA) after centrifugation, and the supernatant was partitioned using ethyl acetate (EtOAc), cyclopentane, and isopropanol (ratio of 49.5:49.5:1, v/v/v). The top organic layer containing free SA was transferred to 4-ml tubes and dried with nitrogen gas. The dried SA was again suspended in 1 ml of 70% methanol. Next, 25 μl of the filtered sample was subjected to HPLC in a C18 reverse-phase HPLC column (HP hypersil ODS, particle size 5 μm, pore size 120 Å Waters; size 3.9 × 300 mm) at a flow rate of 1.0 ml/min (Supplementary Table S1). SA was detected using a Shimadzu fluorescence detector (Shimadzu RF-10AXL), with excitation and emission monitored at 305 and 365 nm, respectively. The quantity of SA was calculated according to the peak value for authentic standards.

For endogenous ABA analysis, 0.3 g of powdered sample was treated with 30 ml of extraction solution (95% isopropanol and 5% glacial acetic acid) and 10 ng of ABA standard [(±)-3,5,5,7,7,7-d6] obtained from the National Research Council of Canada-Plant Biotechnology Institute. The filtrate was concentrated in a rotary evaporator. The residue was dissolved in 4 ml of 1 N sodium hydroxide solution and then washed three times with 3 ml of methylene chloride to remove lipophilic materials. The aqueous phase, brought to a pH value of approximately 3.5 using 6 N hydrochloric acid, was partitioned three times into EtOAc. The EtOAc extracts were then combined and evaporated. The dried residue was dissolved in a phosphate buffer (pH 8.0) and then run through a polyvinylpolypyrrolidone (PVPP) column (Corning Pyrex Borosilicate Glass Chromatography Column with Coarse Fritted Disc, Inner Diameter 10.5 mm × Height 300 mm, 25 ml Capacity). The pH value of the phosphate buffer was adjusted to 3.5 using 6 N HCl, and the sample was partitioned three times into EtOAc. The EtOAc extracts were combined again and evaporated. The residue was dissolved in dichloromethane (CH2Cl2) and passed through a 6 cc silica cartridge (Sep-Pak; Water Associates, Milford, MA, United States) prewashed with 10 ml of diethyl ether/methanol (3:2, v/v) and 10 ml of dichloromethane. The ABA content was recovered from the cartridge by elution with 10 ml of diethyl ether (CH_3_-CH_2_)_2_O: MeOH (3:2, v/v). The extracts were then dried and methylated by adding diazomethane for GC–MS/SIM analysis (6,890 N network GC system and the 5,973-network mass-selective detector, Agilent Technologies, Palo Alto, CA, United States; Supplementary Table S2). For the quantification of ABA, the Lab-Base (ThermoQuest, Manchester, United Kingdom) data system software was used to monitor responses to the ions of m/e 162 and 190 for Me-ABA and 166 and 194 for Me-[2H6]-ABA.

### Histological Staining

The hypersensitive response (HR) of Wt-t and OxF3H-t plants to combined salt and heat stress was compared with that of Wt-cont plants using Trypan blue histological staining and following the method of Koch and Slusarenko (1990). Five leaves were randomly collected from Wt-cont, Wt-t, and OxF3H-t plants after 0, 3, 6, 12, and 24 h of stress exposure, and then, HRs were determined visually. Cell death was quantified using the density of Trypan blue in each leaf. Similarly, the tips and middle of the roots were collected for HR response analysis after 1 week of continuous exposure to combined salt and heat stress. After 3 days of treatment, H_2_O_2_ was detected visually in the leaves using diaminobenzidine (DAB) as a substrate, as reported by Orozco-Cardenas and Ryan (1999). Rice leaves from each treatment group were incubated in DAB (1 mg mL^−1^) for 24 h at 27°C, as described by Chao et al. (2010). Leaves were then destained by boiling in 95% ethanol for 30 min. Ethanol treatment decolorized the chlorophyll in the leaves; only a brown polymerization product, produced by DAB with H_2_O_2_, remained. After boiling, the leaves were cooled at room temperature in ethanol before brown spots were detected.

### Antioxidant Enzyme Assay

Antioxidant enzymes, glutathione peroxidase (GPx), and malondialdehyde (MDA) were measured using the Glutathione Peroxidase Cellular Activity Assay Kit (Sigma) and (MDA) Assay Kit (Sigma), respectively, following the manufacturer’s protocol. About 100 mg of fresh leaves was randomly collected after 0, 3, 6, 12, and 24 h under stress conditions, and these were used to detect the activity of the two enzymes. For the detection of GPx activity, leaves were ground in liquid nitrogen, homogenized in 3 ml of 5% trichloroacetic acid, and centrifuged at 15,000 rpm for 15 min following the method of Bilal et al. (2020). The supernatant was collected in a new 2-mL tube and used for further analysis. A sample, positive control, and blank reaction were used according to the scheme shown in Table 2. Following the user manual, one vial of NADPH assay reagent was reconstituted in 1.25 ml of ddH_2_O. Subsequently, 30 mM of tert-butyl-hydroperoxide was prepared by dilution of 21.5 μl of Luperox TBH70X in 5 ml of ddH_2_O. Then, 250 μl of the prepared reaction mixture, described in Table 2, was placed in a 96-well microplate. The reaction was started by adding 10 μl of 30 mM tert-butyl hydroperoxide. The decline in absorbance at 340 nm was calculated using a wavelength of 340 nm, with an initial delay of 15 s, and an interval of 10 s. Readings were taken six times. The absorbance was calculated in units/mL using the following formula:


$$\Delta A_{340}/6.22\times \mathrm{DF}/V,$$

**Table 2:** Glutathione peroxidase reaction scheme.

	GPx assay buffer (μl)	NADPH assay reagent (μl)	Enzyme (0.25 unit/ml; μl)	Sample (μl)	30 mM t-Bu-OOH (μl)
Blink	940	50	---	---	10
Positive control	900	50	50	---	10
Sample	900	50	---	50	10

where ΔA_340_ = A_340_/min (blank) − A_340_/min (sample); 6.22 = Ɛ^mM^ for NADPH; DF = dilution factor of the sample before adding to the reaction; and V = sample volume in mL.

For malondialdehyde, the kit provided MDA lysis buffer, phosphotungstic acid, BHT 100X, TBA, and MDA standard (4.17 M). The TBA solution was reconstituted by adding 7.5 ml of glacial acetic acid (not provided in the kit), the volume was adjusted to 25 ml by adding ddH_2_O, and the solution was sonicated. A 10-μL measure of 4.17-M MDA solution was diluted with 407 μl of ddH_2_O to prepare 2 mM of standard MDA. Then, 100 μl of diluted solution was added to 900 μl of ddH_2_O to prepare a 0.2-mM MDA solution. Subsequently, 0, 2, 4, 6, 8, and 10 μl of 0.2-mM MDA standard solution was added to a 96-well microplate, and 0- (blank) 0.4-, 0.8-, 1.2-, 1.6-, and 2.0-μL standards were prepared. Thereafter, ddH_2_O was added to each tube to reach a volume of 200 μl. Samples were prepared by homogenizing 10 mg of tissue with 300 μl of MDA lysis buffer containing 3 μl of BHT on ice. The samples were then centrifuged for 10 min at 13,000 rpm after which the residue was discarded. Subsequently, 200 μl of each sample was placed in a 1-ml tube to which 600 μl of TBA was added; this was incubated for 1 h at 95°C and then cooled on ice for 10 min. Finally, 200 μl of the blank and samples was pipetted onto a 96-well microplate, and the absorbance was analyzed at 532 nm. Reaction was run in three technical replicates, and data are calculated using the following formula:


$$S_{a}/S_{v}\times D=C,$$

where S_a_ = amount of MDA in the unknown sample (nmole); S_v_ = sample volume added to each well (ml); D = sample dilution factor; and C = concentration of MDA in the sample.

### Isolation and Quantification of Flavonoids

To quantify flavonoid accumulation in response to combined salt and heat stress, we collected samples after 0, 3, 6, 12, and 24 h under stress conditions. We isolated kaempferol and quercetin from the samples using the protocol described by Neff and Chory (1998) with slight modifications. Approximately 3 g of frozen leaves was ground in liquid nitrogen into a fine powder, homogenized in 30 ml of a methanol and HCl mixture (MeOH:H_2_O:HCl = 79:20:1, v/v/v), and placed on a shaker for 6 h. The crude extracts were then filtered, after which the filtrates were diluted to 2 ml using a rotary evaporator at 30°C and then further dried in a heating block at 60°C overnight. The dried crude extract was dissolved in 1 ml of HPLC-grade ethanol. Reference standards for spectrophotometry were prepared by dissolving 1 mg of each standard sample in 1 ml ethanol. All samples were analyzed in triplicate.

### Sodium and Potassium Accumulation in Plants Under Combined Salt and Heat Stress

The content of Na+ and K+ was determined in all three groups of plants under combined salt and heat combine. Samples were collected after 1 week of continuous stress and crushed into fine powder in liquid nitrogen. From this powder, 0.05 g of samples was digested in 7 ml of 65% NHO3 and 1 ml of 30% H_2_O_2_, before being heated in a microwave (180°C for 20 min), and then allowed to cool for 40 min. The obtained solvent was quantified using inductively coupled plasma mass spectrometry (9ICP-MS; Optima 7900DV, PerkinElmer, United States).

### Chlorophyll Content

After 1 week of continuous exposure to stress conditions, the chlorophyll content was measured in randomly selected leaves using a soil plant analysis development chlorophyll meter (SPAD).

### Statistical Analysis

All experiments were performed in triplicate, and the data from each replicate were pooled. Data were analyzed using two-way ANOVA followed by Bonferroni *post hoc* tests (* shows *p* < 0.05, ** shows *p* < 0.01 and *** shows *p* < 0.001 significant difference) and Duncan’s multiple range test (DMRT). A completely randomized design was used to compare the mean values of different treatments. Data were graphically presented, and statistical analyses were performed using GraphPad Prism software (version 5.01; GraphPad, San Diego, CA, United States) and Statistical Analysis System (SAS 64 bit, United States).

## Results

### F3H Differentially Regulates Rice Phenotypes Under Combined Salt and Heat Stress

**Figure 1:**
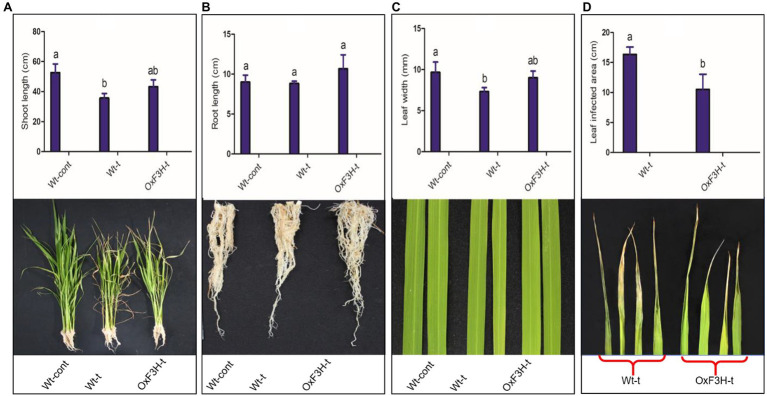
Evaluation of growth parameters of Wt-cont, Wt-t, and OxF3H-t plants. **(A)** Shoot length, **(B)** root length, **(C)** leaf width, and **(D)** leaf tip damage following stress exposure. Lower panels show photographic representations of each phenotype. Different letters above the bars represent significant differences (*p* ≤ 0.001) as determined by Duncan’s multiple range tests.

In this study, we focused on flavanol 3-hydroxylase, which plays a key role in flavonoid biosynthesis and abiotic stress mitigation. We determined the possible role of F3H in plant growth and development by analyzing growth parameters such as root/shoot length, leaf width, and leaf damaged area. The phenotypes under control and stress conditions are shown in Figure 1. The shoot lengths of Wt-t and OxF3H-t plants were significantly different to those of Wt-control plants (Figure 1A); shoot length was reduced by 32 and 17% in Wt-t and OxF3H-t plants relative to Wt-control plants, respectively. Although shoot length was reduced in the transgenic line when compared to Wt-control plants, the reduction was less than that in Wt-t plants, indicating that F3H overexpression is involved in plant growth and development. In contrast, under combined salt and heat stress, no significant difference was found in root lengths among groups, although OxF3H-t roots were 18% longer than those in Wt-control plants (Figure 1B). In addition, we found a significant reduction in leaf width in both Wt-t and OxF3H-t plants when compared with that in the Wt-control plants; however, the leaf width was reduced to a lesser extent in OxF3H plants (6%) than in Wt-t plants (24%; Figure 1C). Heat stress damages leaf tips, which adversely affects plant development and yield; it was previously reported that heat stress reduces leaf area, causes leaf tip burn and yellowing of the entire leaf, and finally leads to death (Sarsu, 2018). Our result showed that the leaf tip was significantly damaged in Wt-t plants when compared with the damage in OxF3H-t plants; we noted about 55% more damage in Wt-t plants (Figure 1D). Beside leaf tip burn, we also noted some necrotic symptoms on the middle area of the leaves of Wt-t and OxF3H-t plants, which were more severe in Wt-t plants (Supplementary Figure S1). Taken together, these results suggest that overexpression of *F3H* mitigates salt and heat stress and can enhance growth and development under stress conditions.

### Overexpression of F3H Enhances the Expression of Salt and Heat Stress-Related Genes Under Combined Salt and Heat Stress

**Figure 2:**
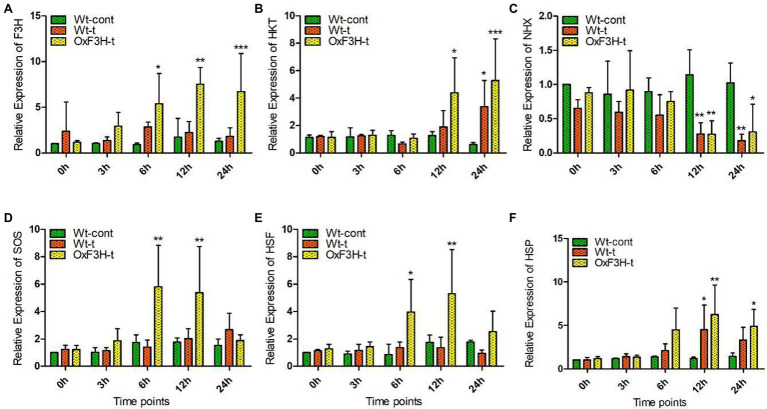
Overexpression of *F3H* significantly regulates salt- and heat-related genes during combined salt and heat stress. **(A–F)** Relative expression of *F3H*, *HKT*, *NHX*, *SOS*, *HSF*, and *HSP*, respectively, in Wt-control, Wt-t, and OxF3H-t plants. The fold change of each gene was measured after 0-, 3-, 6-, 12-, and 24-h intervals of continuous stress exposure, with *actin* used as a reference gene. Graphs show mean ± standard deviation, and asterisks show significant differences (*p ≤ 0.05, **p ≤ 0.01, and ***p ≤ 0.001) according to two-way ANOVA and Bonferroni *post hoc* tests.

To validate whether the 35 s-promoter induced *F3H* gene expression, we assessed the regulation of salt- and heat-responsive genes in response to combined salt and heat stress. We investigated the relative expression level of selected genes after 0, 3, 6, 12, and 24 h of stress exposure (Figure 2). Because the expression of F3H is driven by the promoter, we found consistent expression of *F3H* in the transgenic line and a significant increase in expression after 6, 12, and 24 h of stress exposure (Figure 2A). We found that *HKT* expression increased 248 and 750% in the OxF3H-t line compared with expression in Wt-cont plants after 12 and 24 h, respectively, whereas a 441% increase was found in Wt-t plants after 24 h of stress exposure (Figure 2B). In contrast, *NHX* expression was reduced continually in both Wt-t and OxF3H-t plants, with a significant reduction found in both the plants after 12 and 24 h of stress exposure (Figure 2C). *SOS* and *HSF* showed the same patterns of expression in OxF3H-t plants, and both *SOS* and *HSF* were significantly increased after 6 and 12 h when compared with their expression in Wt-cont plants (Figures 2D,E). However, the expression levels of both genes were reduced after 24 h. We further investigated the expression of *HSP* and found that it was enhanced in both Wt-t and OxF3H-t plants after 6, 12, and 24 h (Figure 2F). However, compared with Wt-cont plants, the expression was significantly different after 12 and 24 h in OxF3H-t plants and after 12 h in Wt-t plants. These results demonstrate that overexpression of *F3H* could differentially regulate salt- and heat-related genes.

### ROS Accumulation and Cell Death in Transgenic and Wild-Type Plants Under Combined Salt and Heat Stress

**Figure 3:**
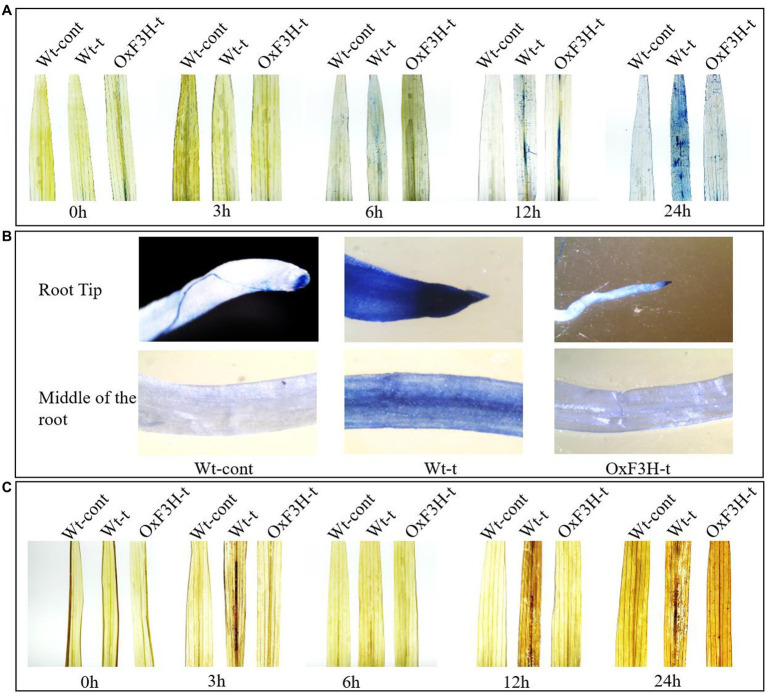
Combined salt and heat stress causes severe oxidative stress in leaves and roots. **(A,B)** Cell death in leaves and roots, respectively, according to Trypan blue staining. **(A)** indicates that substantial cell death occurred in the leaves of Wt-t plants after 12 and 24 h of stress exposure. **(C)** H_2_O_2_ generation in the leaves of Wt-cont, Wt-t, and OxF3H-t plants as detected using DAB staining. Wt-t leaves showed high concentrations of H_2_O_2_ after 12 and 24 h of stress exposure.

ROS are generated as because of common stresses such as wounds, pathogens, drought, salinity, and heat. Beside promoting programmed cell death, high levels of ROS produce harmful effects due to lipid peroxidation, protein denaturation, and DNA damage (Miller and Mittler, 2006). ROS are produced in the form of superoxide anions (O^2−^), hydroperoxide radicals (OH^•^), and hydrogen peroxide (H_2_O_2_). To evaluate H_2_O_2_ generation and cell death, we performed DAB and Trypan blue staining (DAB interacts with H_2_O_2_ and generates a reddish-brown polymer, whereas Trypan blue staining shows dead cells; Hoang et al., 2019). We evaluated the rate of cell death and H_2_O_2_ accumulation in leaves from each of the three plant groups after 0, 3, 6, 12, and 24 h of continuous exposure to combined salt and heat stress. Cell death and H_2_O_2_ accumulation in roots were determined after 1 week of exposure to stress conditions (Figure 3). The leaves of Wt-t plants showed more blue-stained spots compared with those observed on OxF3H-t plant leaves after 12 and 24 h of stress, whereas the Wt-control did not show any staining spots (Figure 3A). Similarly, root tips and the middle parts of the root also accumulated more blue stains compared with that in OxF3H-t plants (Figure 3B); thus, combined salt and heat stress seems to cause substantial levels of cell death in Wt-t plants relative to that in OxF3H-t plants, which may further affect physiological processes. We also noted that the root tip of Wt-t plants, which showed the highest accumulation of blue staining, was more sensitive to the stress condition than that of the OxF3H-t plants. The DAB staining results were consistent with the blue staining; many reddish-brown spots were observed on the Wt-t leaves after 12 and 24 h of stress exposure relative to those on the leaves of OxF3H-t plants (Figure 3C). These results suggest that *F3H* expression is associated with cell death and H_2_O_2_ generation.

### *F3H* Expression Alters Antioxidant and Flavonoid Regulation in Response to Combined Salt and Heat Stress

**Figure 4:**
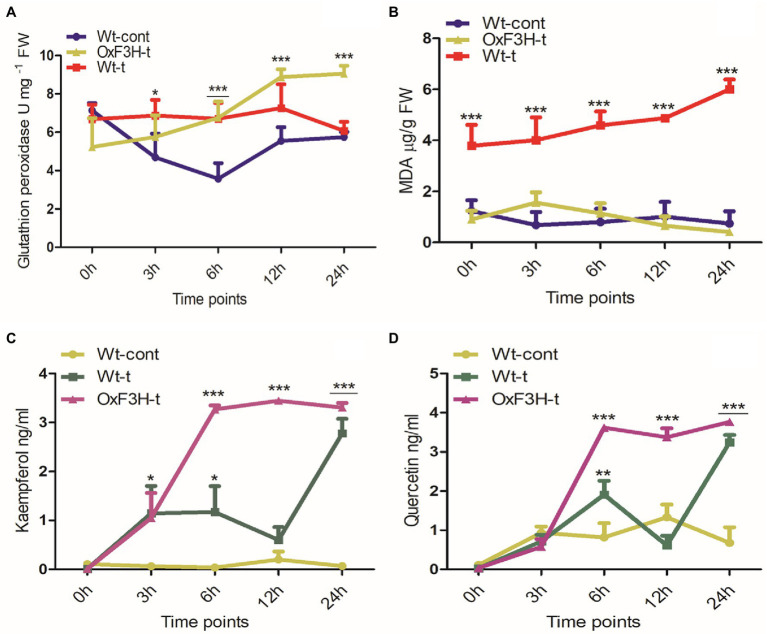
Quantification of antioxidants and flavonoids in wild-type and transgenic plants in response to combined salt and heat stress. **(A)** Glutathione peroxidase, **(B)** lipid peroxidase (MDA), **(C)** kaempferol, and **(D)** quercetin accumulation in Wt-cont, Wt-t, and OxF3H-t plants after 0, 3, 6, 12, and 24 h of continuous exposure to stress conditions. Graphs show the mean ± standard deviation, and asterisks show significant differences (* p ≤ 0.05, ** p ≤ 0.01, and *** p ≤ 0.001) according to two-way ANOVA with Bonferroni *post hoc* tests.

Salt and heat stress regulate osmotic imbalance, which can be adjusted by regulation of flavonoids and antioxidant machinery that ameliorate stress conditions by scavenging free radicals (Kumar et al., 2020). Therefore, we quantified GPx, MDA, kaempferol, and quercetin under combined salt and heat stress after 0, 3, 6, 12, and 24 h of exposure (Figure 4). GPx displayed different patterns of accumulation in control and treated plants during combined salt and heat stress (Figure 4A); GPx was significantly upregulated in OxF3H-t plants compared with in Wt-cont plants, with GPx levels increased by 89, 60, and 57% in OxF3H-t after 6, 12, and 24 h, respectively. However, we noted significant upregulation of GPx in Wt-t plants at 6 h of stress exposure. Compared with OxF3H-t plants, the accumulation of GPx in Wt-cont plants was reduced after 12 and 24 h, indicating that F3H is associated with the regulation of glutathione peroxidase activity. Peroxidation of membrane lipids is a biomarker that characterizes the level of oxidative damage in plants under stress conditions; it is usually measured as an increase in MDA content. We measured MDA in response to combined salt and heat stress was significantly increased in Wt-t plants compared with the levels in Wt-cont plants (Figure 4B). Interestingly, the levels of MDA decreased in the OxF3H-t plants after 12 and 24 h of stress exposure compared with the levels in Wt-control plants. Thus, high levels of membrane lipid peroxidation apparently occurred in Wt-t plants, which led to oxidative damage in these plants. The accumulation patterns of GPx and MDA were antagonistically associated, i.e., GPx increased in OxF3H-t plants, whereas it decreased in Wt-t plants, while MDA levels increased in Wt-t plants and were reduced in OxF3H-t plants. Kaempferol and quercetin showed the same patterns of accumulation in OxF3H-t plants; they increased significantly after 6, 12, and 24 h of stress (Figures 4C,D). In contrast, kaempferol and quercetin showed irregularly accumulation in Wt-t plants compared with that in Wt-cont plants; we noted significantly higher levels of kaempferol after 3 and 6 h of stress exposure, whereas quercetin was significantly increased after 6 h under stress conditions.

### ABA and SA Are Differentially Regulated in Response to Combined Salt and Heat Stress

**Figure 5:**
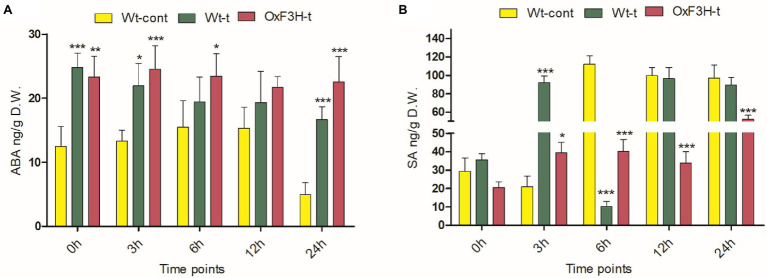
Abscisic acid (ABA) and salicylic acid (SA) are differentially regulated in wild-type and transgenic plants during combined salt and heat stress. **(A)** ABA and **(B)** SA accumulation in Wt-cont, Wt-t, and OxF3H-t plants after 0, 3, 6, 12, and 24 h of continuous exposure to stress conditions. Graphs show means ± standard deviation, and asterisks show significant differences (* p ≤ 0.05, ** p ≤ 0.01, and *** p ≤ 0.001) according to two-way ANOVA with Bonferroni *post hoc* tests.

To investigate the SA and ABA signaling cascade in response to combined salt and heat stress, we quantified their accumulation after 0, 3, 6, 12, and 24 h under stress conditions. ABA levels increased significantly in response to stress exposure in both Wt-t and OxF3H-t plants compared with those in Wt-cont plants (Figure 5A); however, ABA levels were consistently reduced as stress duration increased. Levels of SA were reduced after 6, 12, and 24 h of stress in both the Wt-t and OxF3H-t plants compared with levels in Wt-cont plants (Figure 5B); these levels were significantly lower in OxF3H-t plants. Accumulation of SA was lower in OxF3H-t plants relative to that in Wt-t plants, but SA levels increased as stress duration increased. Comparing the levels of SA and ABA in OxF3H-t plants, the hormones were regulated differentially under stress conditions. Thus, in the *F3H* transgenic line, SA accumulation increased and ABA accumulation decreased in response to combined salt and heat stress.

### Accumulation of Ionic and Chlorophyll Contents Under Combined Salt and Heat Stress

**Figure 6:**
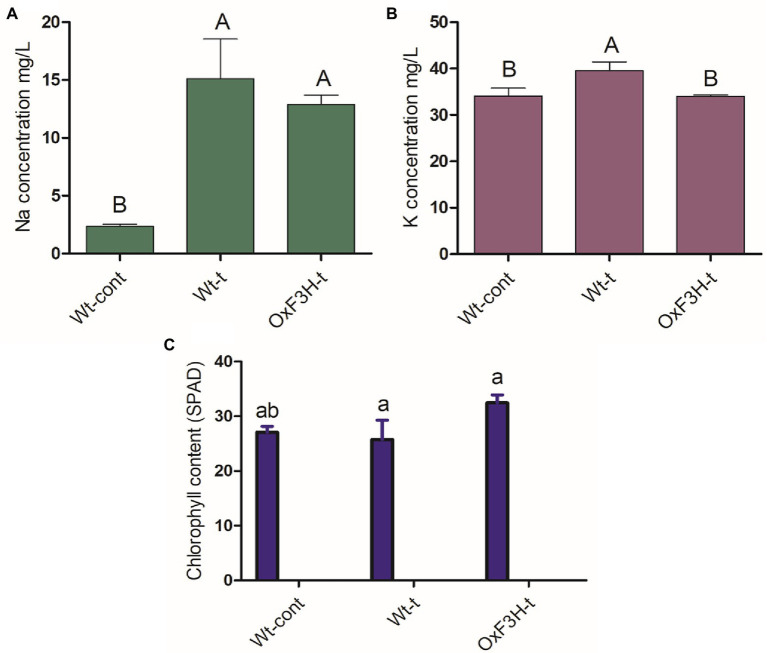
Na+, K+, and chlorophyll accumulation in plants under combined salt and heat stress. **(A)** Na+ concentration, **(B)** K+ concentration, and **(C)** chlorophyll content. Different letters on the bars represent significant differences (p ≤ 0.001) as determined by Duncan’s multiple range tests.

To determine the effects of combined salt and heat stress on Na+, K+, and chlorophyll contents, we measured their concentrations after 1 week of continuous stress exposure (Figure 6). Under stress conditions, Na+ was significantly increased in both Wt-t and OxF3H-t plants compared with levels in Wt-cont plants, with the accumulation of Na+ found to be 14% higher in Wt-t pants than that in OxF3H-t plants (Figure 6A). K+ concentration was also increased (16%) in Wt-t plants relative to that in Wt-cont plants, although K+ levels did not differ between OxF3H-t and Wt-cont plants (Figure 6B). Therefore, the OxF3H transgenic line can tolerate combined salt and heat stress as it accumulates lower levels of Na+ than are accumulated by Wt-t plants and has K+ levels that are similar to those of control plants. Contrastingly, OxF3H-t plants showed the highest level of chlorophyll content of the three plant groups (Figure 6C), indicating that F3H can positively regulate photosynthesis during salt and heat stress.

## Discussion

The purpose of this work was to assess the activity of *F3H* in response to the combined stress of salt and heat, two of the most prominent and damaging abiotic stressors that coexist in arid and semiarid locations across the world. Individually, salt and heat stressors have been extensively investigated, with significant advances made in understanding their related physiological and molecular processes. After validating stress tolerance in the laboratory, researchers can release a substantial number of transgenic crops into agricultural fields. Here, we explored the likely function of flavonoid accumulation in response to combined salt and heat stress, as well as the physiological, biochemical, and molecular responses of plants. The shoot length, root length, leaf width, and leaf tip tolerance levels were all higher in OxF3H-t plants than in Wt-t plants (Figure 1). According to many experts, stress-related inhibition of cell elongation and cell division, irregular ion homeostasis, and osmotic and oxidative stress generated by salt stress could all contribute to decreased plant growth and biomass accumulation (Rahman et al., 2016; Ahmad et al., 2018). In addition to phenotypic variation in wild-type and transgenic lines under stress, we found that OxF3H-t and Wt-t plants had higher and lower photosynthesis levels, respectively, than those in control plants (Figure 6C). According to previous studies, salt stress disrupts photosynthetic pigments in *solanum lycopersicu*m plants, which is associated with oxidative damage (Rahman et al., 2016). Our study indicates that when salt and heat stress are combined, Wt-t plants experience more oxidative stress and ROS accumulation than those observed in OxF3H-t plants, which results in greater chlorophyll degradation in Wt-t plants than in OxF3H-t plants. According to previous observations, oxidative stress stimulates the production of ROS, which enhance chlorophyllase activity, which in turn is responsible for the degradation of photosynthetic pigments (Ahmad et al., 2018). Under combined salt and heat combined stress, OxF3H-t plants produced more kaempferol and quercetin than were produced by Wt-t plants (Figures 4C,D), suggesting that these compounds may be involved in protecting photosynthetic pigments from degradation. Our findings are consistent with those of Parvin et al., (2019), who found that quercetin supplementation enhances photosynthetic pigments in response to salt stress by decreasing the Na+/K+ ratio, scavenging ROS, and lowering osmotic stress. We postulate that increased accumulation of kaempferol and quercetin (Figures 4C,D) detoxifies the damaging H_2_O_2_ molecules produced under combined salt and heat stress due to the antioxidant nature of these compounds.

Beside phenotypic variations, *F3H* overexpression causes transcriptional differences in salt- and heat-related genes in response to combined salt and heat stress. *F3H* is a key gene in the flavonoid biosynthesis pathway, which is upregulated during abiotic stress. In a previous study, we discovered that *F3H* gene expression boosts kaempferol and quercetin accumulation in rice (Jan et al., 2020). It has also been reported that the genes *FLS*, *UFGT*, *F3’H*, and *F3H* are induced in response to drought, UV light, and salt stress and that they enhance the accumulation of flavonoids (kaempferol and quercetin; Martínez-Lüscher et al., 2014; Xu et al., 2020b). These findings suggest that flavonoids and genes involved in their biosynthesis may be implicated in the response to abiotic stressors such as UV radiation, dehydration, heat, and salinity. Under combined salt and heat stress, we measured the expression levels of *HKT*, *NHX*, *SOS*, *HSF*, and *HSP* in both wild-type and transgenic plants (Figure 2). Compared with control plants, *HKT*, *SOS*, *HSF*, and *HSP* were significantly upregulated in OxF3H-t plants, whereas *NHX* was downregulated. Although these genes were expressed at higher levels in Wt-t plants than in control plants, the differences were not statistically significant. Individually, salt stress affects the expression of *HKT*, *NHX*, and *SOS*, but there is little information on how salt and heat stress interact to affect these genes (Zhang et al., 2018). Because *HKT* and *SOS* expression was significantly higher in F3H transgenic plants compared to wild-type and control plants, our findings indicate that F3H enhances *HKT* and *SOS* expression during heat and salt stress. However, based on our findings, we expected that the combination of salt and heat stress suppresses *NHX* expression in OxF3H-t plants compared its expression in control plants. At the protein level, SOS transports Na+ out of the cell, HKT transports internal Na+ into the root xylem, and NHX sequestrates Na+ into vacuole and can relieve sodium toxicity in the stem (Apse et al., 1999; Shi et al., 2002). According to our results, although Na+ and K+ ion levels were higher in Wt-t and OxF3H-t plants compared with the levels in control plants, the accumulation of both was lower in OxF3H-t plants than that in Wt-t plants (Figures 6A,B). Because NHX is involved in the accumulation of Na+ in vacuoles, but its gene expression is reduced in both Wt-t and OxF3H-t plants, either Na+ is not accumulated in the vacuoles during combined salt and heat stress or *NHX* is not expressed under this combined stress. Similarly, in several previous studies, it has been reported that *HSF* and *HSP* play prominent roles in individual salt and heat stresses (Nover et al., 2001; Mittal et al., 2009; Fragkostefanakis et al., 2015). According to our research, *HSF* and *HSP* were upregulated after 6 and 12 h in OxF3H-t plants, but their expression was decreased after 24 h. A previous study showed that heat and salt stress increased accumulation of kaempferol and quercetin *via* regulation of flavonoid biosynthesis-related genes (Xu et al., 2020a). These findings lead us to conclude that F3H, HKT, NHX, SOS, HSF, HSP, kaempferol, and quercetin all work synergistically to increase the tolerance of plants to combined salt and heat stress.

High temperatures and salinity cause oxidative stress, which is mediated by ROS and results in the regulation of stress hormones including ABA, SA, JA, and ethylene. These stress hormones are important for stress mediation and the establishment of a stress–growth balance (Yu et al., 2020). When comparing OxF3H-t to Wt-t and control plants, we found that ABA accumulation was higher, while SA accumulation was lower (Figure 5). Thus, ABA and SA apparently exhibit antagonistic cross talk in response to combined salt and heat stress because SA levels continuously increased, whereas ABA levels decreased as the stress period lengthened. Furthermore, de Torres Zabala et al. (2009) reported that ABA inhibits the synthesis of SA, and Jiang and Zhang (2002) found that ABA triggers the overgeneration of ROS and enhances the antioxidant defense system. Our findings demonstrate that although ABA levels are higher in OxF3H-t plants, ROS accumulation is lower in these plants relative to the levels detected in Wt-t plants (Figure 3). This implies that OxF3H-t plants accumulate more ABA, which in turn lowers oxidative damage during combined salt and heat stress. Furthermore, we discovered that the transgenic line exhibited increased levels of kaempferol, quercetin, and ABA, which suggests that a link exists between flavonoid and hormone signaling in response to combined salt and heat stress. Our findings are consistent with prior research showing that flavonoids were enhanced in ABA-, SA-, and JA-treated plants (Thiruvengadam et al., 2016). Moreover, based on the research of Hung and Kao (2004), we may postulate that ABA and SA regulate the primary enzyme in the flavonoid production pathway, which results in increased levels of kaempferol and quercetin.

## Conclusion

Overall, our study demonstrates that wild-type and OsF3H transgenic rice plants respond differently to combined salt and heat stress in terms of their physiological, biochemical, and molecular responses. During salt and heat stress, induced expression of *F3H* increases plant biomass and photosynthesis. Both salt and heat stress increase oxidative stress, which is mitigated by the high accumulation of kaempferol and quercetin, given that constant expression of *F3H* significantly enhances both of these flavonoid molecules, which are known to scavenge ROS. Furthermore, overexpression of *F3H* upregulates the expression of salt- and heat-related genes, as well as the ionic transport cascade, which is necessary for maintaining the balance of Na+ and K+ ions. Because of the antagonistic cross talk between ABA and SA in one transgenic line, we suggest that *F3H* is involved in the regulation of hormonal machinery in response to combined salt and heat stress. Overall, the overexpression of *F3H* seems to regulate the physiological, biochemical, and molecular machinery of rice plants during stress exposure involving salinity and heat.

## Data Availability Statement

The original contributions presented in the study are included in the article/Supplementary Material; further inquiries can be directed to the corresponding author.

## Author Contributions

RJ, S-HL, and K-MK designed the study. RJ, S-HL, and NK performed the experiments. MK, SAa, and S-HL performed the analyses. I-JL provided resources. RJ and S-HL wrote the manuscript. SAi produced the figures and revised the manuscript. All authors contributed to the article and approved the submitted version.

## Funding

This work was supported by the National Research Foundation of Korea Grant funded by the Korean Government (NRF-2021M3E5E6022715).

## Conflict of Interest

The authors declare that the research was conducted in the absence of any commercial or financial relationships that could be construed as a potential conflict of interest.

## Publisher’s Note

All claims expressed in this article are solely those of the authors and do not necessarily represent those of their affiliated organizations, or those of the publisher, the editors and the reviewers. Any product that may be evaluated in this article, or claim that may be made by its manufacturer, is not guaranteed or endorsed by the publisher.
