# Supplementary material for: Enhanced Flavonoid Accumulation Reduces Combined Salt and Heat Stress Through Regulation of Transcriptional and Hormonal Mechanisms
Source: Front Plant Sci. 2021 Dec 21;12:796956. doi: 10.3389/fpls.2021.796956 (PMC8724123; doi:10.3389/fpls.2021.796956)
Supplement: Supplementary file 1 [file Table_1.pdf]

Supplementary Table S1: HPLC conditions used for analysis and quantification of SA

Equipment	Shimadzu LC-10
Column	HP hypersil ODS (particle size 5μm, pore size 120Å; size 3.9×300mm)
Wavelength	Excitation 305nm, Emission 365nm
Detector	RF-10Axl (fluorescence detector)
Solvent A	100% MeOH
Solvent B	100% water in 0.5% acetic acid
Flow rate	1.0 mL/min
