# Supplementary material for: Enhanced Flavonoid Accumulation Reduces Combined Salt and Heat Stress Through Regulation of Transcriptional and Hormonal Mechanisms
Source: Front Plant Sci. 2021 Dec 21;12:796956. doi: 10.3389/fpls.2021.796956 (PMC8724123; doi:10.3389/fpls.2021.796956)
Supplement: Supplementary file 2 [file Table_2.pdf]

Supplementary Table S2: GC/MS – SIM conditions used for analysis and quantification of ABA

Equipment	Hewlett-Packard 6890, 5973N Mass Selective Detector
Column	HP-1 capillary column (30m×0.25mm i.d. 0.25µm film thickness) (J & W Scientific Co., Folsom, CA, USA)
Carrier gas	He (40 ml/min.); head pressure of 30 kPa
Source temp.	250°C
Oven conditions	ABA : 60°C (1min.) → 15°C/min. → 200°C →5°C/min. → 250°C →10°C /min → 280°C
Injector temp.	200°C
Ionizing voltage	70 eV
