# Supplementary material for: Enhanced Flavonoid Accumulation Reduces Combined Salt and Heat Stress Through Regulation of Transcriptional and Hormonal Mechanisms
Source: Front Plant Sci. 2021 Dec 21;12:796956. doi: 10.3389/fpls.2021.796956 (PMC8724123; doi:10.3389/fpls.2021.796956)

Supplementary Figure S1: Symptoms of combined salt and heat stress on wild-type and transgenic plants.

Wt-cont Wt-t OxF3H-t

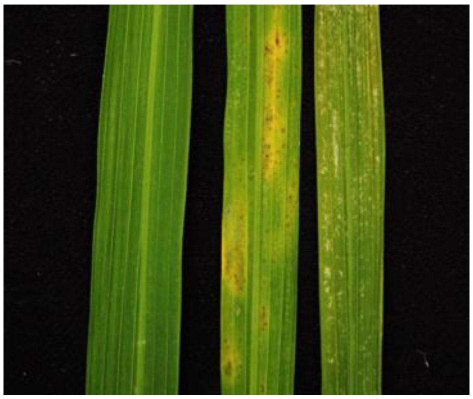

Supplement: Supplementary file 3 [file Image_1.pdf]
